# *Doublesex* Mediates the Development of Sex-Specific Pheromone Organs in *Bicyclus* Butterflies via Multiple Mechanisms

**DOI:** 10.1093/molbev/msaa039

**Published:** 2020-02-20

**Authors:** Anupama Prakash, Antónia Monteiro

**Affiliations:** m1 Department of Biological Sciences, National University of Singapore, Singapore; m2 Yale-NUS College, Singapore

**Keywords:** *doublesex*, scent organs, sex-specific trait development, androconia, *Bicyclus* butterflies, ancestral state reconstruction

## Abstract

The *Bicyclus* lineage of satyrid butterflies exhibits male-specific traits, the scent organs, used for chemical communication during courtship. These organs consist of tightly packed brush-like scales (hair-pencils) that rub against scent patches to disperse pheromones, but the evolution and molecular basis of these organ’s male-limited development remains unknown. Here, we examine the evolution of the number and location of the scent patches and hair-pencils within 53 species of *Bicyclus* butterflies, and the involvement of the sex determinant gene *doublesex* (*dsx*) in scent organ development in *Bicyclus anynana* using CRISPR/Cas9. We show that scent patches and hair-pencils arose via multiple, independent gains, in a correlated manner. Further, an initially nonsex-specific Dsx protein expression pattern in developing wing discs becomes male-specific and spatially refined to areas that develop the scent patches. Functional perturbations of *dsx* show that this gene activates patch development in males whereas hair-pencils develop in both sexes without Dsx input. Dsx in females is, instead, required to repress hair-pencils whereas Dsx in males regulates minor aspects of its development. These findings suggest that the patches and hair-pencils evolve as correlated composite organs presumably due to their functional integration. Divergence in the function of *dsx* isoforms occurred across the sexes, where the male isoform promotes patch development in males and the female isoform represses hair-pencil development in females, both leading to the development of male-limited traits. Furthermore, evolution in number of patches in males is due to the evolution of spatial regulation of *dsx.*

## Introduction

Diverse sex-specific traits are present in many animal lineages, arising as products of natural or sexual selection acting predominantly on one sex versus the other. Examples of such traits include variation in body size, color, exaggerated morphologies, or behaviors and physiologies that aid each sex in acquiring more mates and/or in producing a larger number of offspring (Jonsson and Alerstam 1990; Gross 1996; Shine 1989). The enormous diversity and spectacular nature of some of these sex-specific traits combined with their important ecological and adaptive functions has promoted multiple studies looking at the evolution and development of such traits (Williams and Carroll 2009; Kopp 2012; Rogers et al. 2013; Gotoh et al. 2014; Neville et al. 2014).

The origin of sexually dimorphic traits is especially intriguing from a genetic perspective and was intensely debated by Darwin and Wallace (Kottler 1980). The two men debated whether novel sex-specific traits can arise in a single-sex from the very beginning or whether novel traits always arise first in both sexes but are then lost in one to create sexual dimorphisms. In addition, each supported the importance of different selective forces in producing such dimorphisms. Wallace strongly believed that natural selection was key to converting equally inherited, showy traits to male-limited traits for the protection of females, because females were under stronger selection by predators, whereas Darwin argued that traits could arise in only one sex (primarily in males) from the very beginning and be further amplified via sexual selection (Kottler 1980). This debate is only now being resolved with the use of sex-specific reconstructions of traits on phylogenies which help reconstruct single-sex or dual-sex origins of traits as well as their subsequent evolution (Emlen et al. 2005; Kunte 2008; Oliver and Monteiro 2011). While it appears that both modes of evolution occur in a variety of taxa, the genetic and developmental mechanisms that allow the development of traits in one sex but limit their occurrence in the other sex, are still largely unresolved for most sexually dimorphic traits.

**Fig. 1.**
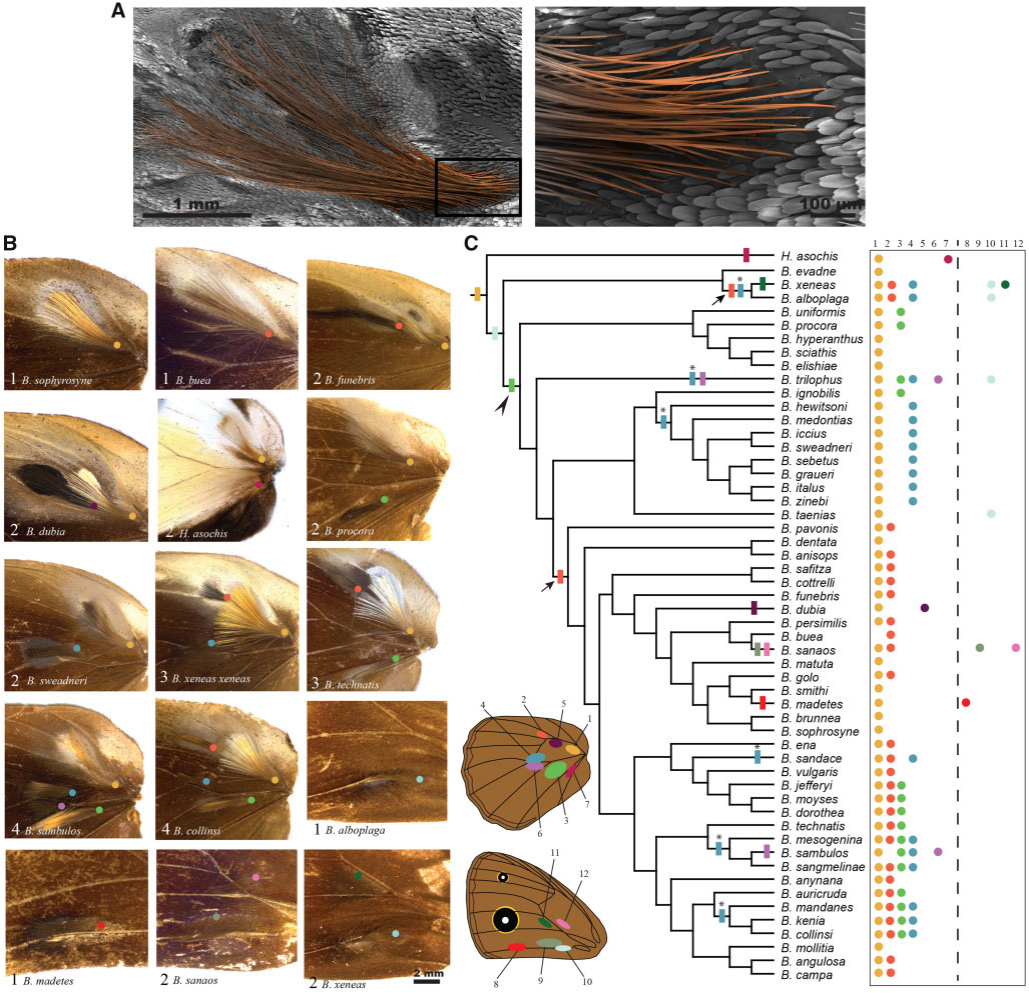
Diversity and evolution of hair-pencils on the wings of *Bicyclus* butterflies. (*A*) Artificially colored scanning electron micrograph of the hindwing, yellow hair-pencil (hair-pencil 1) in *Bicyclus anynana*. The boxed area (base of the hair-pencil scales) is expanded to the right to illustrate the elongated and densely packed nature of the hair-likes scales that make up a hair-pencil. (*B*) The different combinations of hindwing and forewing hair-pencils in male *Bicyclus* butterflies. The number of hair-pencils and the name of the species are denoted at the bottom left. Colored circles indicate the position of the base of each hair-pencil corresponding to the schematic in *C*. (*C*) Evolutionary history of hair-pencils at homologous positions on the wing within the *Bicyclus* lineage. Locations, number, and color codes of the different traits are given in the schematic and their presence/absence in each species is listed to the right. Filled rectangles on the phylogenetic tree indicate the likely gains of the traits for which either a single- or a multiple-origin scenario at the MRCA of all the species bearing that trait was significantly supported. Arrows indicate the two likely gains for hair-pencil 2 and stars indicate the six likely gains for hair-pencil 4 within *Bicyclus*. For traits where a single- or multiple-origins scenario was equally supported, a single-origin was mapped (black arrowhead).

**Fig. 2.**
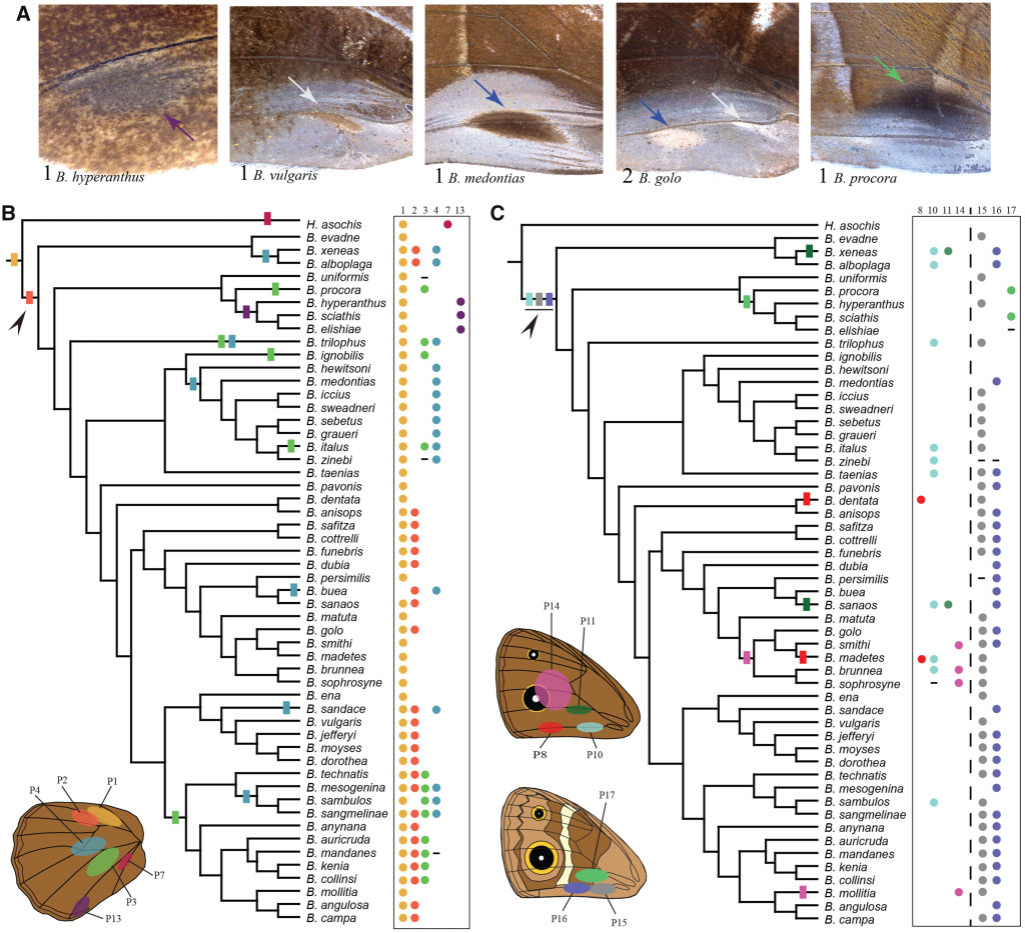
Diversity and evolution of patches on the wings of *Bicyclus* butterflies. (*A*) From left: An example of patch 13 on the dorsal hindwing and combinations of patches 15, 16, and 17 on the ventral forewing of male *Bicyclus* butterflies. Note the broad area of silvery scales near the posterior margin of the ventral forewings. The number of patches and the name of the species are denoted at the bottom left. Colored arrows point to each patch and correspond to the colors used in the schematics in *B* and *C*. For examples of hindwing patches, refer to figure 1*B*. (*B*, *C*) Evolutionary history of (*B*) hindwing and (*C*) forewing scent patches at homologous positions on the wing within the *Bicyclus* lineage. Locations, number and color codes of the different patches are given in the schematic and their presence/absence in each species is listed to the right of the phylogenetic trees. Species for which data were not available are marked with “–.” Color codes of the patches match the codes of the corresponding hair-pencils in those sectors. Filled rectangles on the phylogenetic tree indicate the likely gains of the traits for which either a single- or a multiple-origin scenario at the MRCA of all the species bearing that trait was significantly supported. For traits where a single- or multiple-origins scenario was equally supported, a single-origin was mapped (black arrowheads).

The scent organs in butterflies are a clear example of a sexually dimorphic, male-specific trait used for close-range, premating chemical communication. These composite organs, collectively called androconia, differ dramatically in shape, color, pheromone composition and location, occurring as complexes of scent patches with secretory glands and overlying modified epidermal scales that help produce and release pheromones, and tightly packed, brush-like hair-pencils on the legs, abdomen, thorax, and wings of butterflies that help disperse these pheromones during courtship (Boppre 1989; Birch et al. 1990; Doerge 2002; Hall and Harvey 2002; Hernández-roldán et al. 2014). In some species, some of the structures in the complex develop on different parts of the body and require special behaviors to ensure contact (Boppre 1989). For example, many male butterflies in the tribe Danaini (family Nymphalidae) have extrusible, brush-like abdominal hair-pencils that are brought into physical contact with pheromone producing patches on the wings to enable pheromone dissemination (Boppre 1989). On the other hand, many species in the tribe Satyrini possess scent organs consisting of hair-pencils (fig. 1*A*, *B*) and scent patches (fig. 2*A*), both on the wing, that brush against each other dispersing chemicals in the process (Nieberding et al. 2008; Brattstr-m et al. 2015, 2016; Aduse-Poku et al. 2017). It is to be noted, however, that glandular, secretory cells underly some of the patches and produce pheromones, whereas other patches are not associated with such secretory cells (Bacquet et al. 2015; Dion et al. 2016).

Research into chemical ecology has spurred several studies on the chemical composition of pheromones, their plasticity and their evolution across the genus *Bicyclus* (Nymphalidae, Satyrinae, Satyrini) (Nieberding et al. 2008, 2012; Heuskin et al. 2014; Bacquet et al. 2015; Dion et al. 2016; Darragh et al. 2017; Balmer et al. 2018). However, no study to date has addressed the development and evolution of these sex-specific complexes. The male-specific androconia of *Bicyclus* butterflies are so diverse and variable in number, size, shape, color, chemical composition and position that they are a key trait in species identification (Condamin 1973; Brattstr-m et al. 2015, 2016). The frequent changes in the morphology and location of the two components of the androconia—the hair-pencils and scent patches—within the *Bicyclus* clade, combined with their presence in the model species *Bicyclus anynana*, makes this a unique study system to understand the evolutionary history and, dissect the molecular and developmental genetic mechanisms governing male-specific pheromone complex development.

We used a combination of phylogenetic mappings and a focused molecular investigation on the sex-determination gene, *doublesex* (*dsx*), to address these questions. Our focus on *dsx* was due to two main reasons. Firstly, previous work on sex-specific trait development in *B. anynana* identified a non-cell-autonomous, hormonal mechanism as a determinant of sex-specific eyespot sizes (Bhardwaj et al. 2018). Here, sex-specific levels of the hormone 20-hydroxyecdysone and the presence of its receptor EcR cued the development of different dorsal eyespot sizes in males and females (Bhardwaj et al. 2018) but showed no effect on the development of the male-specific scent organs, indicating a different mechanism of determination of this sexually dimorphic trait. This study also showed that *dsx* was expressed in these scent organs but not in eyespots. Secondly, in the rapidly increasing literature of sexually dimorphic trait development across insects, *dsx* appears to hold a highly conserved position in directing sex-specific gene expression that results in sex-specific morphologies and behaviors, cell-autonomously or in concert with hormones to incorporate environmental cues (Rideout et al. 2010; Kopp 2012; Gotoh et al. 2014; Prakash and Monteiro 2016). The *dsx* gene is spatially regulated in different somatic tissues such that only a subset of cells express *dsx* and are sex-aware (Robinett et al. 2010). It exhibits a modular genetic architecture, where modules evolve under different selective pressures, suggestive of functional partitioning between the different modules over evolutionary time (Baral et al. 2019). Moreover, distinct isoforms of *dsx* are produced in each sex, creating a sex and tissue-specific transcription factor asymmetry that can be used to direct cells into distinct developmental fates (Burtis and Baker 1989; Ito et al. 2013; Neville et al. 2014). Thus, evolution in patterns of spatial and temporal regulation of *dsx* (Tanaka et al. 2011), of its downstream targets (Ledón-Rettig et al. 2017), or in the mode of action of the different isoforms, that is, as repressors or activators (Kopp et al. 2000; Kijimoto et al. 2012; Arbeitman et al. 2016), are all previously investigated mechanisms that can create vastly different, dynamically evolving, sex-specific morphologies in different insect lineages.

In this study, we first independently mapped the presence of hair-pencils and scent patches at homologous positions onto a phylogeny of the *Bicyclus* clade to understand the evolution of these traits. Then, we studied the role of *dsx* in the development of the sexually dimorphic scent organ complex in *B. anynana* using immunostainings and targeted gene knockouts using CRISPR/Cas9. Finally, we examined an outgroup species, *Orsotriaenae medus* (Nymphalidae, Satyrinae, Satyrini), that diverged from *Bicyclus* nearly 40 Ma, to estimate the degree of conservation of Dsx expression in scent organ development across satyrids.

## Results

### Androconia Diversity within *Bicyclus*

Males in the genus *Bicyclus* display enormous diversity in the number and position of the hair-pencils (fig. 1*B*) and scent patches on their wings (fig. 2*A*). Hair-pencils are present only on the dorsal surfaces of both wings (fig. 1*C*, schematic) with the total number of forewing hair-pencils (0–2) being equal to or less than the number of hindwing hair-pencils (1–4) in any given species. Scent patches occur on all wing surfaces except the ventral hindwings (fig. 2*B*, *C*, schematic). As with the hair-pencils, forewing scent patches are rarer (0–2 per surface) (fig. 2*C*) as compared with hindwing scent patches (1–4 per surface) (fig. 2*B*). In most cases, hair-pencils are usually associated with an underlying patch (indicated by the same colors in figs. 1 and 2) but there are instances of hair-pencils present without any associated patch (hair-pencil 3 without patch 3 in *Bicyclus jefferyi*, *Bicyclus moyses*, and *Bicyclus dorothea*) and vice versa (patch 13 in *Bicyclus hyperanthus*, *Bicyclus scaithis*, and *Bicyclus elishiae*).

### Patterns of Androconia Diversity within *Bicyclus* Occurs Primarily via Multiple Trait Gain and Trait Loss

In order to understand the origins of this diversity within *Bicyclus*, we first constructed a phylogenetic tree of the species of interest using a Bayesian framework and then reconstructed the evolutionary history of the different hair-pencils and patches on the sampled trees. Our majority-rule Bayesian consensus tree (supplementary fig. 1, Supplementary Material online) was largely congruent with the Bayesian tree published in Aduse-Poku et al. (2017). We obtained the *Bicyclus* clade as a well-supported monophyletic group and the *evadne*-species group as a sister clade to all other *Bicyclus*, similar to the earlier published data. However, as with the Aduse-Poku et al. (2017) tree, some of the basal branches had poorer support giving rise to some basal uncertainty, which was accounted for in our ancestral state reconstructions.

We reconstructed the evolutionary history of the different hair-pencils and scent patches on the sampled trees. Ancestral state reconstruction of the hair-pencil present at the base of the discal cell, what we call hair-pencil 1, indicated that this trait is ancestral and was present before the divergence of the *Bicyclus* clade, with one loss in *Bicyclus buea* (fig. 1*C*, yellow rectangle, supplementary table 2, Supplementary Material online), with a similar situation for the associated patch 1 (fig. 2*B*, yellow rectangle). Hypothesis tests for forewing hair-pencil 10 provided significant support for the model where the trait was present in the most recent common ancestor (MRCA), similar to hair-pencil and patch 1 (fig. 1*C*, light blue rectangle, supplementary table 2, Supplementary Material online) followed by multiple losses. For hair-pencils 2, 4, and 6 (fig. 1*C*, orange, blue, and lilac) and patches 3 and 4 (fig. 2*B*, green and blue), hypothesis tests comparing alternative reconstructions of trait origin within *Bicyclus*, that is, the definitive presence versus the definitive absence of the trait at the MRCA of all the species bearing the trait, provided significant support toward the model where the trait was absent in the MRCA (supplementary table 2, Supplementary Material online). Significant support for MRCA = 0 was also obtained for dorsal forewing patches 8, 11, and 14 (fig. 2*C*, red, dark green, and pink). This supports a scenario of multiple, independent gains of the different traits during the evolution of *Bicyclus* with subsequent losses in many lineages.

To broadly understand the likely number of independent gains of these traits, we used stochastic character mapping and an unconstrained model of trait evolution to identify gains at internal nodes (figs. 1*C* and 2*B*, *C*; rectangles). For example, hair-pencil 2 was likely gained at least twice—once in the *evadne*-species group and once after the branching off of the *ignobilis-hewitsoni* group (fig. 1*C*, orange rectangles and arrows). Similarly, hair-pencil 4 was likely gained six times within *Bicyclus* (fig. 1*C*, blue rectangles, and stars). However, for a few traits including hindwing hair-pencil 3 (fig. 1*C*), hindwing patch 2 (fig. 2*B*), dorsal forewing patch 10 (fig. 2*C*), and ventral forewing patches 15 and 16 (fig. 2*C*) there was no definitive support for one state over the other at the MRCA, making the reconstruction of the evolution of these traits ambiguous (supplementary table 2, Supplementary Material online). For such traits, we adopted the most parsimonious evolutionary explanation of a single-origin at the MRCA (figs. 1*C* and 2*B*, *C*; black arrowheads). Trait losses are not mapped on the phylogenetic trees.

### Scent Patches and Hair-Pencils Appear to Evolve in a Correlated Manner

We next tested for correlated evolution between pairs of hair-pencils and patches. Hair-pencils 1–4 and patches 1–4, respectively, showed strong support for dependent evolution (supplementary table 3, Supplementary Material online) consistent with our understanding of a hair-pencil and a patch together making a functional composite scent organ for pheromone dissemination. In addition, there was positive support for correlated evolution between hair-pencil 1 and patch 15 and, very strong support for correlated evolution between hair-pencil 2 and patch 16 (supplementary table 3, Supplementary Material online). Patches 15 and 16, which are located on the ventral forewing, are in contact with hair-pencils 1 and 2 on the dorsal hindwing, respectively at the region of overlap between the two wings. The evidence for correlated evolution between these forewing patches and hindwing hair-pencils are again suggestive of a composite scent organ, now dispersed between two different surfaces.

### Dsx Expression Changes from Monomorphic to Sex-Specific in the Presumptive Scent Organ Wing Regions over the Course of Development

**Fig. 3.**
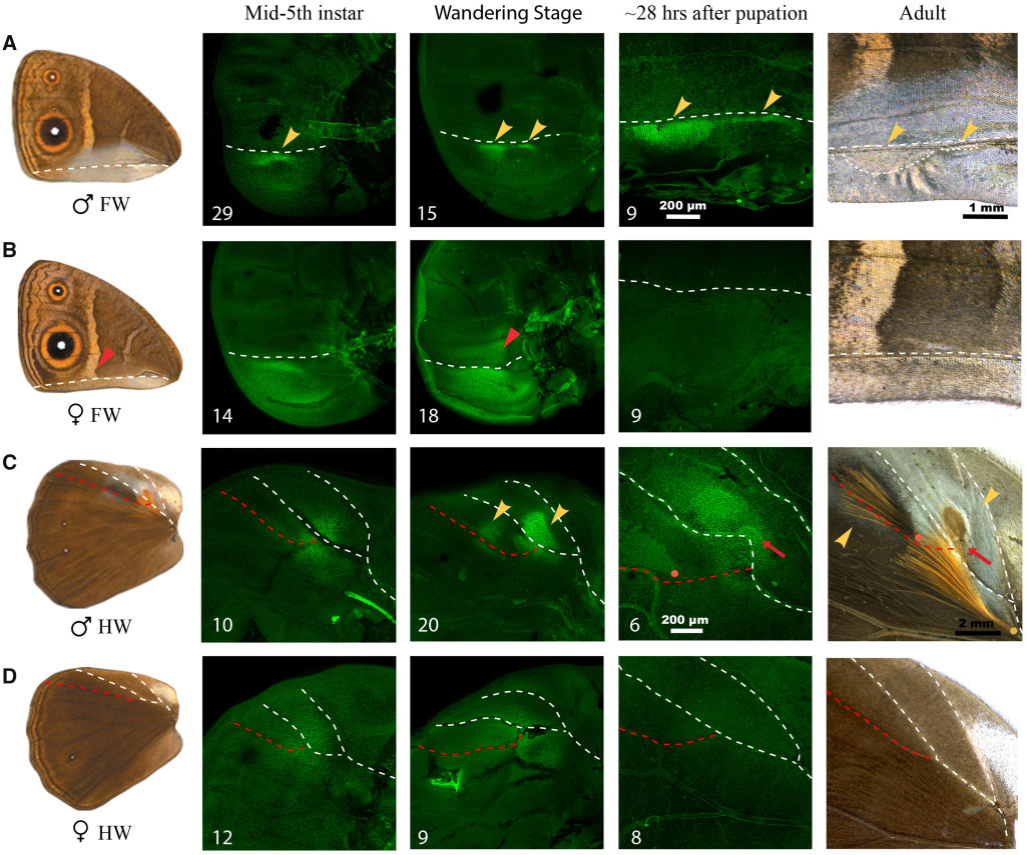
Dsx expression during wing development in *Bicyclus anynana*. Dsx expression during three different wing developmental stages, that is, midfifth instar, wandering stage and 28 h after pupation are shown for (*A*) male and (*B*) female forewings and (*C*) male and (*D*) female hindwings. The adult wings are shown in the first column and an expanded view of the scent organ regions in both sexes is shown in the last column. Images shown are the best, illustrative images and numbers at the bottom left indicate sample size. White and red dotted lines denote homologous veins in the respective wings and the orange and yellow dots in (*C*) correspond to the base of hair-pencils as in figure 1. Yellow arrowheads in (*A*) and (*C*) indicate the two scent patches on the forewing and hindwing, respectively and the red arrows in (*C*) indicate the secretory cells at the center of the patch. Scale bars are shown for the pupal wings and adult structures.

To identify the proximate mechanisms for male-specific androconia development, we examined the expression of the sex-determinant protein Dsx in developing wing discs of both sexes in *B. anynana*. Adult male *B. anynana* butterflies possess dorsal hindwing hair-pencils 1 and 2 with their corresponding patches, and ventral forewing patches 15 and 16. Secretory cells lie beneath dorsal hindwing patch 1 and ventral forewing patches 15 and 16 (Dion et al. 2016). In addition, males also possess a broad area of silvery scales near the posterior margin of the ventral forewing. Dsx was expressed at low levels throughout the forewing and hindwing discs of both sexes with strong Dsx expression mapping to the general scent patch and hair-pencil regions early in wing development (fig. 3—midfifth instar, supplementary fig. 3, Supplementary Material online). As development progressed, this strong pattern of Dsx showed sex-specific variation in the presumptive scent organ regions: from the last larval instar (fifth), through the wandering stage, to the pupal stage (fig. 3, supplementary figs. 3–5, Supplementary Material online). The prepupal stage was not conducive to any sort of staining, and inferences about the role of Dsx at this stage were based on Dsx expression in the preceding and succeeding developmental stages. Forewing and hindwing Dsx expression patterns are described below.

The Dsx expression pattern in forewings became associated with the development of the scent patches and silver scales in males. In midfifth instar forewing discs, Dsx was strongly expressed in sector 1A + 2A in both sexes (fig. 3*A*, *B*) (sector and vein nomenclature in supplementary fig. 2, Supplementary Material online), with a narrow linear expression in the anterior part of the sector in males (fig. 3*A*; below the white dotted line—yellow arrowhead). In the following wandering stage, expression diverged across the sexes. In males, the single, linear expression of Dsx was defined into two domains, corresponding to the future locations of scent patches 15 and 16, which was further refined into precise spatial domains, resembling the shapes of the two patches, ∼28 h after pupation (fig. 3*A*; yellow arrowheads). At this pupal stage Dsx expression was also present in punctate nuclei that mapped to the general region of the ventral forewing silver scales in males. In females, however, expression of Dsx remained broad within the proximal sector 1A + 2A at the wandering stage, expanding into the region of the white band in the Cu2 sector (fig. 3*B*; red arrowhead). By 28 h after pupation, no strong Dsx expression was seen mapping to a particular structure in the corresponding wing regions (fig. 3*B*).

The hindwing discs also showed a similar progression of Dsx expression over time where the initial broad association of Dsx with the location of both scent patches and hair-pencils in both sexes became more refined and restricted in male wings only. In both male and female midfifth instar discs, there was a circular region of heightened Dsx expression at the proximal end of sector Rs, extending partially into the discal cell (fig. 3*C*, *D*), which potentially covered both the scent patch and hair-pencil domains. This broad expression domain was later refined into two spatial regions in male wandering stage discs corresponding to the male hindwing scent patches with surrounding silver scales (patch 1) and, the patch of grayish-silver scales that lie below the future black hair-pencils (patch 2) (fig. 3*C*; yellow arrowheads). No strong Dsx expression was seen in the presumptive hair-pencil regions at this stage. About a day after pupation, male hindwing expression of Dsx was spatially refined and modified, now marking the secretory cells underneath patch 1 (fig. 3*C*; red arrows) and the hair-pencils (fig. 3*C*). In the hair-pencil cells however, Dsx expression was not strongly localized to the nucleus (fig. 3*C*, supplementary fig. 5, Supplementary Material online) unlike the scent patches. As mentioned previously, secretory cells underly certain patches in *Bicyclus* species, including *B. anynana*, but not all patches. In females, the initial expression of Dsx continued in the wandering stage but by 28 h after pupation there was no Dsx expression associated with a particular structure in corresponding regions of the female hindwing discs (fig. 3*D*). Control images and co-immunostainings of Dsx with DAPI for wings at the midfifth instar, wandering and pupal stages for both sexes are presented in supplementary figures 3–5, Supplementary Material online.

### Sex-Specific Isoforms of *Dsx* Regulate the Development of the Two Components of the Scent Organ in Different Ways

To identify if *dsx* was sex-specifically spliced in the male and female wing tissues of *B. anynana*, *dsx* was amplified and sequenced from the wings. Male and female pupal wing discs expressed different isoforms of *dsx* (supplementary fig. 6, Supplementary Material online) suggesting that *dsx* is indeed sex-specifically spliced in the wing tissues. The partial sequences generated are available on GenBank (accession nos. MK869725 and MK869726).

**Fig. 4.**
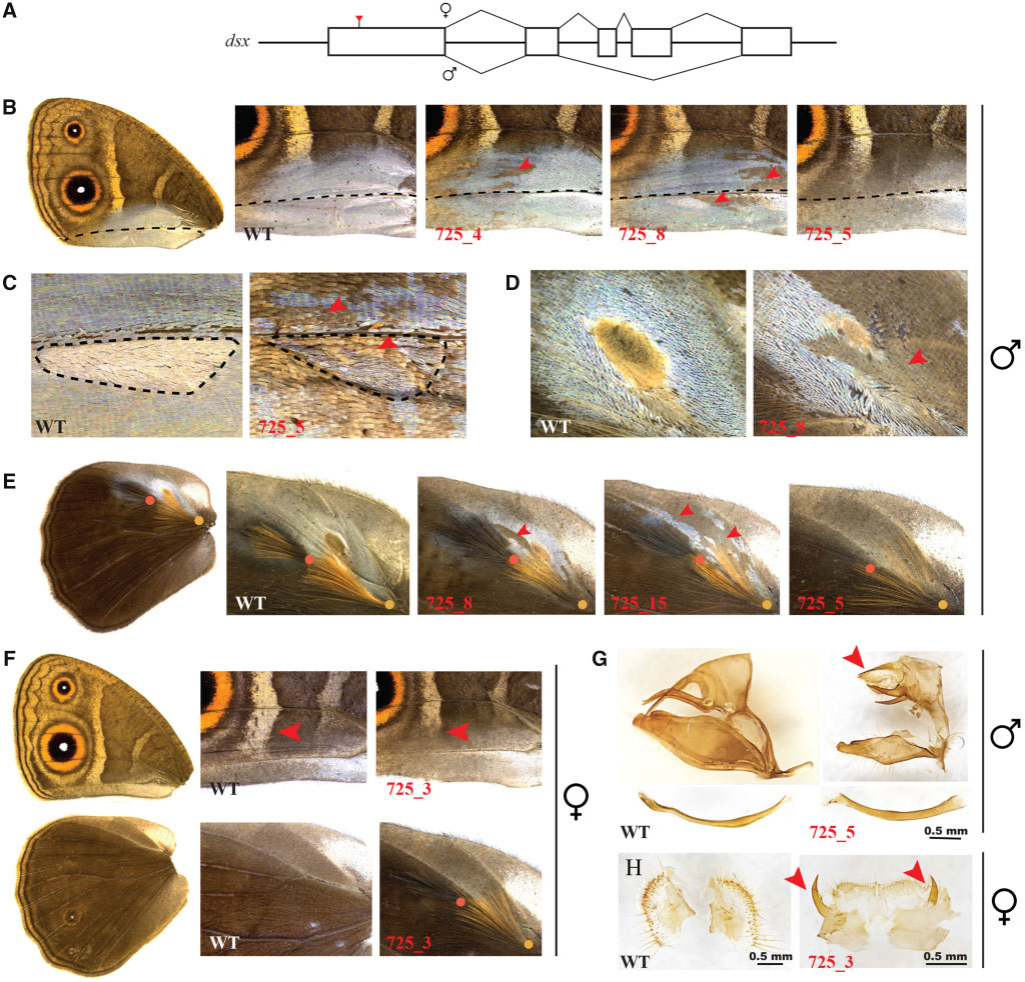
CRISPR/Cas9 directed *dsx* crispant phenotypes in *Bicyclus anynana*. (*A*) Schematic of *dsx* male and female isoforms with the region targeted by CRISPR/Cas9 marked with a red arrow. In panels *B*–*H*, wildtype (WT) is shown to the left and crispants are indicated in red to the right. (*B*–*E*) Mosaic phenotypes on male (*B*, *C*) ventral forewing patches and broad silvery scale region and (*D*, *E*) dorsal hindwing patches. Black dotted lines in (*B*) highlight homologous veins and in (*C*) outlines patch 16. Red arrowheads indicate some of the mosaic phenotypes. (*F*) *dsx* crispant phenotypes on female ventral forewings (top) and dorsal hindwings (bottom). Red arrowheads indicate the differences in length of the white band between WT and crispant. (*G*) Intersex phenotypes of male genitalia with reduced claspers, aedeagus (bottom) and an intermediate morphology of the uncus (red arrowhead). (*H*) Intersex phenotypes of female genitalia. Intermediate structures are indicated with red arrowheads.

To functionally verify the role of *dsx* in the development of the male-specific scent organs in *B. anynana*, we used CRISPR/Cas9, targeting the common DNA binding domain, which is shared between the male- and female-specific isoforms (fig. 4*A*). In males, knockout of *dsxM* affected the development of both forewing and hindwing scent patches and surrounding silver scales, corresponding to Dsx’s region of expression in developing wing discs. For both wings, crispants showed varying degrees of loss of silver scales (fig. 4*B*–*E*; red arrowheads) and scent patches with secretory cells (fig. 4*C*, *D*, supplementary fig. 7*A*, *B*, Supplementary Material online; black arrows). These results suggest an activating role for *dsxM* in males, in the development of the scent patches and the broad silvery scale region. *dsxM* knockout in males also reduced the density of hair-like scales of both hair-pencils in many individuals (supplementary fig. 7*F*, Supplementary Material online) but none of the crispants showed absence of either hair-pencil despite the loss of the silvery scales that lie adjacent to them, indicating effective disruption of *dsx* in that general wing area (supplementary fig. 7*C*, Supplementary Material online; black arrowheads).

Knockout of *dsxF* in females showed contrasting results on its effect on the two androconia components. Crispant females developed pairs of dorsal hair-pencils on either both or only one hindwing (fig. 4*F*), suggesting that *dsx* in females represses hair-pencil development. However, these hair-pencils had, on average, a lower density of hair-like scales compared with wildtype and crispant males (supplementary fig. 7*F*, Supplementary Material online). None of the female crispants displayed any scent patches and associated silver scales on either forewings or hindwings, indicating the lack of a role of *dsxF* in repressing the development of these scent organ components. Additionally, on the ventral forewings, *dsx* crispant females showed modifications to the white transversal band that now stopped midway into the CuA2 sector instead of extending all the way to vein 1A + 2A as in wildtype (fig. 4*F*; red arrowheads). This indicates that *dsxF* promotes the development of the posterior band in females. None of the *dsx* crispants showed any effects on eyespot size.

#### *dsx* Affects the Morphology of the Genitalia

*dsx* crispant males and females also developed deformed genitalia, displaying intersex phenotypes. The aedeagus and claspers in males became reduced in size, whereas the main body of the genital structure, the uncus, displayed an intermediate morphology (fig. 4*G*; red arrowhead). In females, the two sclerotized genital plates also developed intersex structures (fig. 4*H*). The sex of crispants was verified by amplifying a microsatellite that occurs on the female-limited W chromosome (supplementary fig. 7*E*, Supplementary Material online) and effective *dsx* disruptions were confirmed by sequencing DNA from thoracic tissue (supplementary fig. 8, Supplementary Material online). Injection statistics and quantification of the different *dsx* crispant phenotypes is provided in supplementary tables 5 and 6, Supplementary Material online.

### Scent Organ Associated Dsx Expression Arose before the Divergence of the *Bicyclus* Lineage

**Fig. 5.**
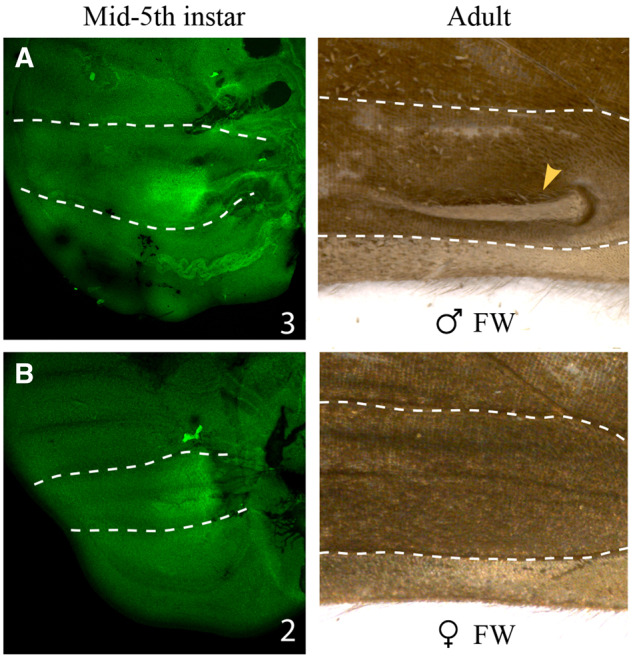
Dsx expression in developing forewing discs of *Orsotriaena medus*. Dsx expression (left) in midfifth instar forewing discs of (*A*) males and (*B*) females. Corresponding adult ventral wing regions are shown to the right. Note the extruded scent patch present only in adult male wings (yellow arrowhead). Images shown are the best, illustrative images and numbers at the bottom right indicate sample size. White lines indicate homologous veins in the respective wings.

To verify if *dsx*-mediated development of androconia in *B. anynana* and, potentially all other *Bicyclus* species predated the origin of the genus, we performed Dsx antibody stainings on midfifth instar forewing discs of a distantly related satyrid species, *O. medus*, displaying male-specific scent organs. Males of this species possess a pocket-like scent patch on the dorsal forewings with an associated hair-pencil. This pocket appears as an extruded portion of the wing on the ventral forewing surface (fig. 5*A*, Adult; yellow arrowhead indicating the extruded wing patch on the ventral forewing). Both male and female midfifth instar forewing discs showed spatial expression of Dsx in the presumptive scent organ regions (fig. 5). This could map to either hair-pencil or patch in males but is unclear at this time because we did not look at surface-specific expression of Dsx, that is, dorsal or ventral expression. Nevertheless, these observations, similar to those in *B. anyanna*, suggest that scent organ associated Dsx expression likely originated before the divergence of the *Bicyclus* and *Orsotriaena* lineages.

## Discussion

### Patterns of Androconia Evolution within *Bicyclus*

Our phylogenetic analyses suggest that androconia have extremely labile evolutionary histories, with frequent gains and losses of both scent patches and hair-pencils—a pattern of evolution not unlike that of dorsal eyespots, which are also traits used in sexual signaling (Robertson and Monteiro 2005; Prudic et al. 2011), and which are also extremely labile within *Bicyclus* (Oliver et al. 2009)*.* Most of the traits investigated were absent in the common ancestor to all *Bicyclus*, except for hair-pencil 1 at the base of the discal cell on the hindwings and its corresponding patch 1, which are both old and evolutionarily stable. Most other hair-pencils and patches were gained multiple times during the evolution of this genus. This pattern suggests that the gene regulatory networks (GRNs) that build hair-pencils and patches were kept intact throughout the *Bicyclus* radiation, because one or more of these traits were always present somewhere on the wings, however, GRN redeployments and losses took place multiple times during evolution. Evolution in the trait’s location on the wing and in trait number might perhaps be achieved via tinkering with the expression of master regulator genes of either GRN, still to be discovered. Thus the evolution of the composite scent organs in *Bicyclus* show evolutionary trends similar to loss and regain of other complex characters such as eyespots (Oliver et al. 2009) or shell coiling in gastropods (Collin and Cipriani 2003).

Additionally, hair-pencils and patches also showed correlated evolution across *Bicyclus*. This makes sense considering that the two components together form a functional composite organ required for pheromone production and dissemination in some species such as *B. anynana*. However, many species possess only one of these functionally coupled traits which begs the question of their functional roles in these species. Patches without correspondent hair-pencils were more commonly present than hair-pencils without correspondent patches. Examples for both include the presence of velvety patches on the dorsal forewings and hindwings of members of the *martius*- and *sciathis*-groups, respectively (fig. 2, patches 13 and 14), or the presence of hair-pencil 3 and the absence of corresponding patch 3 in *B. jefferyi*, *B. moyses*, and *B. dorothea* (figs. 1*C* and 2*B*). Despite the possibility of patches being functional in chemical communication, independently of associated hair-pencils, we note that many patches in *Bicyclus* do not secrete any pheromones (Bacquet et al. 2015). Thus, the functionality of these organs and their role, or lack of it, in the behavioral biology of *Bicyclus* butterflies still remains an open question. Their functions in chemical communication might have been lost over evolutionary time or these organs might now function in visual communication instead (Bacquet et al. 2015).

Since our analyses were limited to the *Bicyclus* clade, it is still unclear how many other lineages possess similar wing scent organs, and where (which lineage and which position on the wing) the first hair-pencil and patch serial homolog originated. A more detailed and inclusive phylogenetic sampling across all lineages with scent organs would be needed to determine the origins of these traits and compare their evolutionary patterns with what we identified in the *Bicyclus* clade.

### Precise and Refined Spatial Expression of Dsx Determines Sex-Specific Scent Organs

Investigation of the proximate mechanisms governing male-specific scent organ development in *B. anynana*, identified two different modes of action of the sex-specific *dsx* isoforms in determining the two components of the organ. Precise spatial expression of Dsx on male wings preceded the development of male-specific scent patches. This expression domain, initially broad and non-sex-specific, was refined during development to map precisely to the final adult male structures. Dsx expression was also visualized in the silvery scale region on the ventral forewings at early stages of pupation. Our functional data indicate that DsxM is required for the development of the male-specific scent patches and the silvery scales, as disruption of *dsxM* led to the loss of both traits in males. In contrast, hair-pencils develop in both sexes when either *dsxM* or *dsxF* is disrupted. This suggests that DsxF is involved in repressing hair-pencil development in females and that DsxM is not involved in the determination of hair-pencils in males, as females don’t have this *dsx* male isoform and yet develop hair-pencils (upon disruption of *dsxF*). Disruptions to *dsx*, however, led to lower density of hair-like scales in both crispant males and females compared with wildtype, but none of the male crispants had either hair-pencil missing despite the loss of the silvery scales and patches 1 and 2 that lie in the immediate vicinity of the hair-pencils (fig. 4*E*, supplementary fig. 7*C*, Supplementary Material online; black arrowheads), indicating effective disruption of *dsx* in this wing area. These results suggest that DsxM may have a smaller role in up-regulating hair-pencil density in males and DsxF a role in down-regulating density in females.

**Fig. 6.**
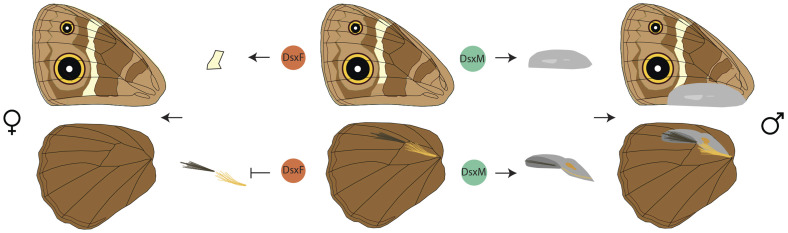
Schematic of the effects of sex-specific *dsx* isoforms on *Bicyclus anynana* wing patterns and sex-specific scent organs. Centre shows the wing phenotype of ventral forewing (top) and dorsal hindwing (bottom) in the absence of either *dsx* isoform. Dsx in males activates the development of patches on both fore and hindwings and regulates hair-pencil density (right). Dsx in females increases the length of the white band toward the posterior end of the forewing and represses hair-pencils on the hindwing (left).

Our results also suggest that the two components of the scent organ in *B. anynana* are determined at different developmental stages via modulation of an initially non-sex-specific expression of Dsx, and that the sex-limited presence of this organ is governed by two different modes of *dsx* action: *dsxM* acting as an activator of scent patches and as a regulator of hair-pencil density in males and *dsxF* acting as a repressor of hair-pencils in females (fig. 6). In female wings, the expression of Dsx in the general area of the hair-pencils early in wing development, rather than in the precise location of the hair-pencils, could be sufficient for its repressive function. On the other hand, spatially refined Dsx expression patterns in female wings may have been present in the prepupal stage, before hair-pencils are formed, which could not be investigated in this study due to the inability to stain prepupal wing discs. Such distinct modes of action of *dsx* isoforms have been previously identified in *Drosophila melanogaster* (Goldman and Arbeitman 2007; Arbeitman et al. 2016) as well as in other species. For example, in the beetle *Onthophagus taurus*, the male isoform of *doublesex* promotes head horn development whereas the female isoform represses horn development in females (Kijimoto et al. 2012). In contrast, in a closely related species, *Onthophagus sagittarius*, that exhibits a reversed sex-specific thoracic horn phenotype, the male *doublesex* isoform inhibits horn development whereas the female isoform promotes its growth (Kijimoto et al. 2012). Furthermore, in the case of *Drosophila* abdominal pigmentation, although the male isoform has a negligible effect on pigmentation, the female isoform represses such pigmentation in females (Kopp et al. 2000).

The sex-specific development of the scent patches is determined by the spatial regulation of *dsx*, in a similar way to the regulation of *dsx* in the sex-combs in *Drosophila* (Tanaka et al. 2011). Spatial regulation of *dsxM* is required for the precise development of the male-specific patches whereas potentially less precise spatial regulation of *dsxF* is required for the repression of the hair-pencils in females, leading to a male-limited occurrence of this trait. The refinement of Dsx expression in males suggests the involvement of other spatially restricted transcription factors in the regulation of *dsx* over time, similar to the *Scr*-*dsx* feedback loop involved in *Drosophila* sex-comb development (Tanaka et al. 2011). Candidate transcription factors that might be involved in dorsal surface-specific regulation of *dsx* include *apterous A* and factors downstream of *apterous A* (Prakash and Monteiro 2018), whereas ventral-specific factors are unknown. In addition, the downstream targets of *dsx* in the development of the scent organs still remain unknown and constitute an interesting avenue for future research.

Further, our identification of scent organ associated Dsx expression in the more distantly related satyrid butterfly *O. medus* suggests that *dsx*-mediated sex-specific androconial development arose before the divergence of these two clades. It is then highly plausible that the proximate mechanisms we have identified here in *B. anynana* are applicable to other *Bicyclus* species, with the male-specific *dsx* isoform required only for patch development in males whereas the female *dsx* isoform is required to repress hair-pencils in females. This hypothesis, however, requires additional functional work in the other *Bicyclus* species.

### Different Proximate Mechanisms Generate Male-Specific Structures on the Same Wing Surface

Our results show two different mechanisms governing the development of sex-specific traits within one species, bearing resemblance to both Darwin and Wallace’s views on the origin of sexual trait dimorphism. The finding that *dsxM* leads to activation of scent patches in males only, lends support to Darwin’s theory of a trait being able to arise in a single-sex right from the very beginning. This is because, this protein, only found in male bodies, promotes the development of a complex male trait. On the other hand, the finding that *dsxF* represses hair-pencil development in females, suggests that the hair-pencil GRN might have initially originated in both sexes, but was subsequently removed from females using a protein that is only found in female’s bodies, resembling Wallace’s origins of sexually dimorphic traits. However, we acknowledge that both the Darwin and Wallace views on the origins of sexual trait dimorphisms, in this particular case of scent organ origins, would need further testing by functional verification in other *Bicyclus* species.

### Molecular Mechanisms of Scent Organ Development and the Evolution of Androconia Diversity

Given our understanding of the proximate mechanisms of sex-specific scent organ development in one *Bicyclus* species, and the patterns of trait evolution within this genus, we can draw certain inferences and propose hypotheses about the mechanisms of androconia evolution within the *Bicyclus* clade. Since spatial regulation of the male *dsx* isoform is required for patch development, diversification in the number of patches could have occurred via the gain and loss of new domains of *dsx* expression on the wing. However, the diversity in hair-pencils cannot be explained via the acquisition and loss of new domains of *dsx* because hair-pencil development can occur independently of *dsx*. Instead, we hypothesize that the origin of the hair-pencil GRN probably arose via the deployment of the GRN that determines long hair-like scales, in a dense cluster of neighboring cells. These long scales are morphologically like hair-pencils and occur at low density on the wings of many butterflies of both sexes, even in families that do not possess hair-pencils (supplementary fig. 9, Supplementary Material online). The hair-pencil GRN could then have been modified via DsxM mediated inputs and co-opted to different locations on the wing, generating diversity in number and morphology with sex-specificity being created by DsxF-mediated repression of this network in females. Thus, in the particular case of hair-pencils, spatial regulation of *dsx* does not lead to diversity in the number of sex-specific traits, it leads solely to the creation of sex-specificity of these traits, a situation that differs from that of sex-comb evolution in *Drosophila* (Tanaka et al. 2011).

An additional possibility for the diversity in scent organs observed could be due to introgression or the process of gene transfer between closely related species because of hybridization. Wing pattern mimicry between closely related *Heliconius* species has previously been explained by adaptive introgression (The Heliconius Genome Consortium 2012; Smith and Kronforst 2013; Zhang et al. 2016) and such a mechanism could also explain diversity in androconia within *Bicyclus*, where many species occur in sympatry in Africa. Identifying the “on” and “off” switches of key genes in the GRNs that create hair-pencils and patches and, performing comparative molecular studies can help validate these hypotheses and also potentially explain the correlated evolution between the two traits.

In conclusion, this study, concurrent with the study on sex-specific eyespot development in *B. anynana* (Bhardwaj et al. 2018), have together identified three different ways of producing sexual dimorphisms in one species via both cell-autonomous and non-cell-autonomous mechanisms—activation of the scent patches only in males via DsxM, female-specific repression of hair-pencils via DsxF (both acting through cell-autonomous mechanisms) and, a third, non-autonomous hormonal threshold mechanism controlling sex-specific eyespot sizes. This diversity in proximate mechanisms within one organism has implications for the origins and rapid turnover of sexually dimorphic traits and must be considered while exploring the evolution of sex-specific traits.

## Materials and Methods

### Animal Husbandry

Mated *O. medus* females were collected in forested areas of Singapore under the permit number NP/RP14-063-3a. *Bicyclus anynana* and *O. medus* butterflies were reared in a 27 °C temperature-controlled room with 65% humidity and a 12:12 h light:dark photoperiod. Adults of both species were fed on banana. *Bicyclus anynana* larvae were fed on young corn plants whereas *O. medus* larvae were fed on *Ischaemum* sp. grasses, commonly found in Singapore.

### Character Sampling and Phylogenetic Analyses

Fifty-three *Bicyclus* species listed in the phylogeny of Monteiro and Pierce (2001) and 1 outgroup were considered for this study, however, we used the larger set of 179 individuals (95 *Bicyclus* species and 10 outgroups), 10 gene data set from Aduse-Poku et al. (2017) to reconstruct a Bayesian phylogenetic tree using MrBayes v3.2.7a (Ronquist et al. 2012). This reconstruction allowed us to account for phylogenetic uncertainty in the ancestral state reconstruction of the androconia. Nucleotide sequences were aligned based on the translated amino acid sequences and then concatenated to create a data set of 7,704 characters from 10 genes. The partitioning scheme provided by Aduse-Poku et al (2017) was used to partition this data set. Two parallels of four chains (three heated and one cold) were run on MrBayes for 5 million generations and a split frequency below 0.01 was used to assess stationarity. Burn-in was set to 25% (2,500 trees) and the remaining set of trees from both runs were pooled together to total 15,000 trees for the following analyses. The postburn-in trees were also used to produce a majority-rule consensus tree. These trees were then pruned in Mesquite (Maddison and Maddison 2018) to only include the 54 taxa used in the Monteiro and Pierce study, which we had in hand and could examine for the presence and location of the male scent organs.

For character sampling, hindwings and forewings of the different *Bicyclus* and outgroup species were imaged using a Leica DMS 1000 microscope. The images in combination with data from Condamin (Condamin 1973) and other scientific literature (Nieberding et al. 2008; Bacquet et al. 2015; Brattstr-m et al. 2015, 2016; Aduse-Poku et al. 2017) were used to score the presence and absence of hair-pencils and scent patches in each species. We defined a hair-pencil as a group of tightly packed brush-like scales. On the dorsal hindwing and dorsal forewing, we defined a scent patch as an area of modified epidermal scales, different from the background color. We did not examine the presence or absence of secretory cells beneath the patches. On the ventral forewing, *Bicyclus* species usually possess a broad area of silvery scales near the posterior margin of the wing. Within this region there are usually one to two smaller areas of modified epidermal scales, most often associated with secretory cells underneath. We scored these smaller regions as scent patches on the ventral forewing, similar to the scoring scheme in Bacquet et al. (2015). In cases where two scent patches were located within sector 1A + 2A (see sector and vein nomenclature in supplementary fig. 2, Supplementary Material online) on the ventral forewing, they were distinguished based on their position to the left or right side of a hypothetical perpendicular line drawn from the intersection of veins M3 and CuA1 to the vein 1A + 2A. Hair-pencils and patches were considered homologous between species if they occupied similar positions within the same sector on the wing, that is, a region bound by the wing veins. The character matrix is provided in supplementary table 1, Supplementary Material online.

### Ancestral State Reconstruction

Visualizations of the ancestral state reconstructions of the different traits was carried out in Mesquite (Maddison and Maddison 2018) using the majority-rule consensus tree. A two-parameter asymmetric model of evolution was used to allow for different rates of gain and loss of hair-pencils and patches.

For a more rigorous statistical hypothesis testing of the origins of the different traits within *Bicyclus*, ancestral states were also reconstructed across the posterior distribution of trees generated in MrBayes (15,000 trees) using a reverse jump MCMC method as implemented in the MULTISTATE package in BayesTraits V3 (http://www.evolution.rdg.ac.uk/BayesTraitsV3.0.1/BayesTraitsV3.0.1.html; last accessed May 2019) (Pagel et al. 2004). Reverse jump MCMC accounts for both phylogenetic uncertainty and the uncertainty in estimation of parameters of the model for trait evolution during the ancestral state reconstruction. To test whether a particular state, that is, 1 (trait present) or 0 (trait absent), was statistically supported for each trait at the MRCA of all lineages bearing the trait of interest, model marginal likelihoods were calculated with each alternative state fixed at the MRCA. Phylogenetic hypothesis testing was done by comparing the log marginal likelihoods of the two models and a model was considered significantly more likely if the log Bayes Factor = 2*Δ log marginal likelihood, was >2 (Pagel 1999). When the trait was present at the MRCA of the species bearing it, then it was considered homologous, otherwise, it was considered analogous (with multiple origins). All RJ-MCMC chains were run for 5 million generations with a burn-in of 25% and a uniform prior between 0 and 100. The marginal likelihoods of the different models were estimated using 500 stones of a stepping stone sampler as per the BayesTraits manual. Further, to map the gains of traits that were significantly supported as either homologous or not within *Bicyclus*, we ran an unconstrainted model of trait evolution and estimated the probable ancestral states at internal nodes. We also ran stochastic character mapping in Mesquite and used both the analyses to broadly understand the evolution of hair-pencils and patches in *Bicyclus*.

Correlations between pairs of traits was estimated using the program DISCRETE in BayesTraits (Pagel and Meade 2006), with a RJ-MCMC run across the 15,000 postburn-in trees and the same parameters as mentioned above. Dependence between two traits were investigated by comparing the log marginal likelihoods of an independent model (traits evolve independently) versus a dependent model (traits evolve in a correlated manner) and a log Bayes Factor = 2*(log marginal likelihood [dependent model] – log marginal likelihood [independent model]) > 2 was considered a significant support toward the dependent model.

### Dsx Immunostainings

We used a primary monoclonal antibody (mouse) raised against the *Drosophila* Dsx protein DNA binding domain (DsxDBD; Developmental Studies Hybridoma Bank deposition by Baker, B.S.) (Mellert et al. 2012) that is present in both male and female isoforms of Dsx. The DsxDBD antibody staining of male wandering stage wing discs correlated with previously published in situ hybridization stains of *dsx* at the same stage (Bhardwaj et al. 2018), suggesting that the signals we detected using this antibody accurately represent Dsx protein expression. This antibody was also previously used to detect sex-specific Dsx expression on *Agraulis vanilla* (Martin et al. 2014) and *Papilio polytes* wing discs (Kunte et al. 2014). Alexa Fluor 488-conjugated donkey anti-Mouse antibody (Jackson ImmunoResearch Laboratories, Inc.) was used as a secondary antibody.

Wing discs were dissected from both sexes at different stages during development and transferred to cold fix buffer (0.1 M PIPES pH 6.9, 1 mM EGTA pH 6.9, 1% Triton X-100, 2 mM MgSO_4_). Pupal wings were transferred to fix buffer at room temperature and moved onto ice after addition of fixative to prevent crumpling of the tissue. Fixation was in 4% formaldehyde (added directly to the wells) for 30 min on ice, followed by five washes with PBS. Peripodial membrane was not removed for larval wings. The wings were then transferred to block buffer (50 mM Tris pH 6.8, 150 mM NaCl, 0.5% IGEPAL, 5 mg/ml BSA) overnight at 4 °C. Incubation with primary antibody at a concentration of 2.5 µg/ml in wash buffer (50 mM Tris pH 6.8, 150 mM NaCl, 0.5% IGEPAL, 1 mg/ml BSA) was for 1 h at room temperature, followed by four washes (20 min each) with wash buffer. Wings were subsequently incubated in secondary antibody (1:500) diluted in wash buffer for 30 min at room temperature and then washed with wash buffer twice, followed by an incubation with DAPI (1:1,000) for 5 min. Wings were then washed with wash buffer four times (10 min each) and immediately mounted onto slides with mounting media. Samples were imaged on an Olympus FLUOVIEW FV3000 confocal microscope.

### CRISPR/Cas9 Gene Editing

Cas9-mediated gene editing in *B. anynana* followed the protocol in Prakash and Monteiro (2018). Briefly, Cas9 mRNA was obtained by in vitro transcription of linearized pT3TS-nCas9n plasmid (a gift from Wenbiao Chen [Addgene plasmid #46757]) using the mMESSAGE mMACHINE T3 kit (Ambion) and tailed using the Poly(A) Tailing Kit (Ambion) following the manufacturer’s protocol. Guide RNA targets were manually designed by looking for GGN_18_NGG sequence in *dsx* exons, preferably targeting a protein domain. sgRNA templates were prepared according to Bassett et al. (2013) and purified templates were in vitro transcribed using T7 RNA polymerase (Roche). 900 ng/µl of purified Cas9 mRNA and 400 ng/µl of purified guide RNA were mixed along with blue food dye and injected into eggs within 2 h of egg laying. Injections were done with a Borosil glass capillary (World Precision Instruments, 1B100F-3) using a Picospritzer II (Parker Hannifin). Hatched caterpillars were reared on fresh corn leaves and emerging adults were scored for their phenotypes (supplementary tables 5 and 6, Supplementary Material online). Mutated individuals were tested for indels at the targeted site by genomic DNA extraction from thoracic tissues, amplification of targeted regions, cloning and sequencing. All primers and guide RNA sequences are listed in supplementary table 4, Supplementary Material online.

### Genitalia Dissections

The genitalia from wildtype and crispants were dissected using fine forceps and placed in 10% solution of sodium hydroxide for 30 min to 1 h to soften the attached nonsclerotized tissues. They were then moved to PBS and the sclerotized structures were separated from the underlying tissues using forceps. Genitalia were embedded in low melting agarose to maintain orientation while imaging. Imaging was done using an Ocellus microscope (Dun Inc.) consisting of a Canon 7D Mk II DSLR body, Mitutoyo objectives and a P-51 Camlift stacking rail. Individual image slices were processed with Lightroom (Adobe Inc.), stacking of images with Zerene Stacker and postprocessing with Photoshop CS5 (Adobe Inc.).

## Supplementary Material

msaa039_Supplementary_Data
