# Supplementary material for: Doublesex Mediates the Development of Sex-Specific Pheromone Organs in Bicyclus Butterflies via Multiple Mechanisms
Source: Mol Biol Evol. 2020 Feb 20;37(6):1694–707. doi: 10.1093/molbev/msaa039 (PMC7253200; doi:10.1093/molbev/msaa039)
Supplement: msaa039_Supplementary_Data [file msaa039_supplementary_data.zip › msaa039-suppl_data/Supplementary Figures and Tables.pdf]

Supplementary Materials for

Doublesex mediates the development of sex-specific pheromone organs in *Bicyclus* butterflies via multiple mechanisms

Anupama Prakash* and Antónia Monteiro*

correspondence to: anupama@u.nus.edu or antonia.monteiro@nus.edu.sg

Supplementary Materials and Methods

Identifying *doublesex* isoforms in wings

Total RNA was extracted from ~18 hr pupal wings of males and females using TRIzol reagent (Invitrogen) according to the manufacturer's protocol. cDNA was prepared by reverse transcription using the RevertAid First Strand cDNA Synthesis Kit (Thermo Scientific, USA). The canonical male and female *dsx* isoform sequences in *Bicyclus anynana* were kindly provided by Arjen Van't Hof. *dsx* transcritps were amplified from the wing tissues of both sexes using primers designed against sequences that are common to both male and female isoforms (Supplementary Table 4). PCR amplifications were done using 2x PCRBIO Taq Red Mix (PCR Biosystems) with the following conditions: 1 min at 96°C, 40 cycles of 15s at 96°C, 15s at 60°C and 10s at 72°C.

Sexing adults

A W-microsatellite PCR-based method was used to verify the sex of the crispants (van't Hof *et al.* 2005). The primer sequences that amplify a 185 bp product present only on the W chromosome, were kindly provided by Arjen Van't Hof and are listed in Supplementary Table 4. PCR was carried out in a 12.5µl reaction volume using 2x PCRBIO Taq Red Mix (PCR Biosystems) and the following protocol: 3 min at 95°C, 40 cycles of 30s at 95°C, 30s at 60°C and 45s at 70°C. Only females produce a band while males do not.

Counting hair-pencil scales and statistical analysis

Images of hair-pencils from 20 crispant males, 12 crispant females and 10 wildtype individuals were captured using a Leica DMS 1000 microscope. A glass slide was used to flatten and spread the hair-pencil scales to make it easy to count. Differences between the number of hair-pencil scales was tested using a one-way ANOVA in R Studio (RStudio Team 2019). Pairwise comparisons were done using a post-hoc Tukey's HSD test, adjusting for multiple comparisons.

References:

RStudio Team. (2019). RStudio: Integrated Development Environment for R.
van't Hof, A.E., Zwaan, B.J., Saccheri, I.J., Daly, D., Bot, A.N.M. & Brakefield, P.M.

(2005). Characterization of 28 microsatellite loci for the butterfly Bicyclus anynana. *Mol. Ecol. Notes*, 5, 169–172.

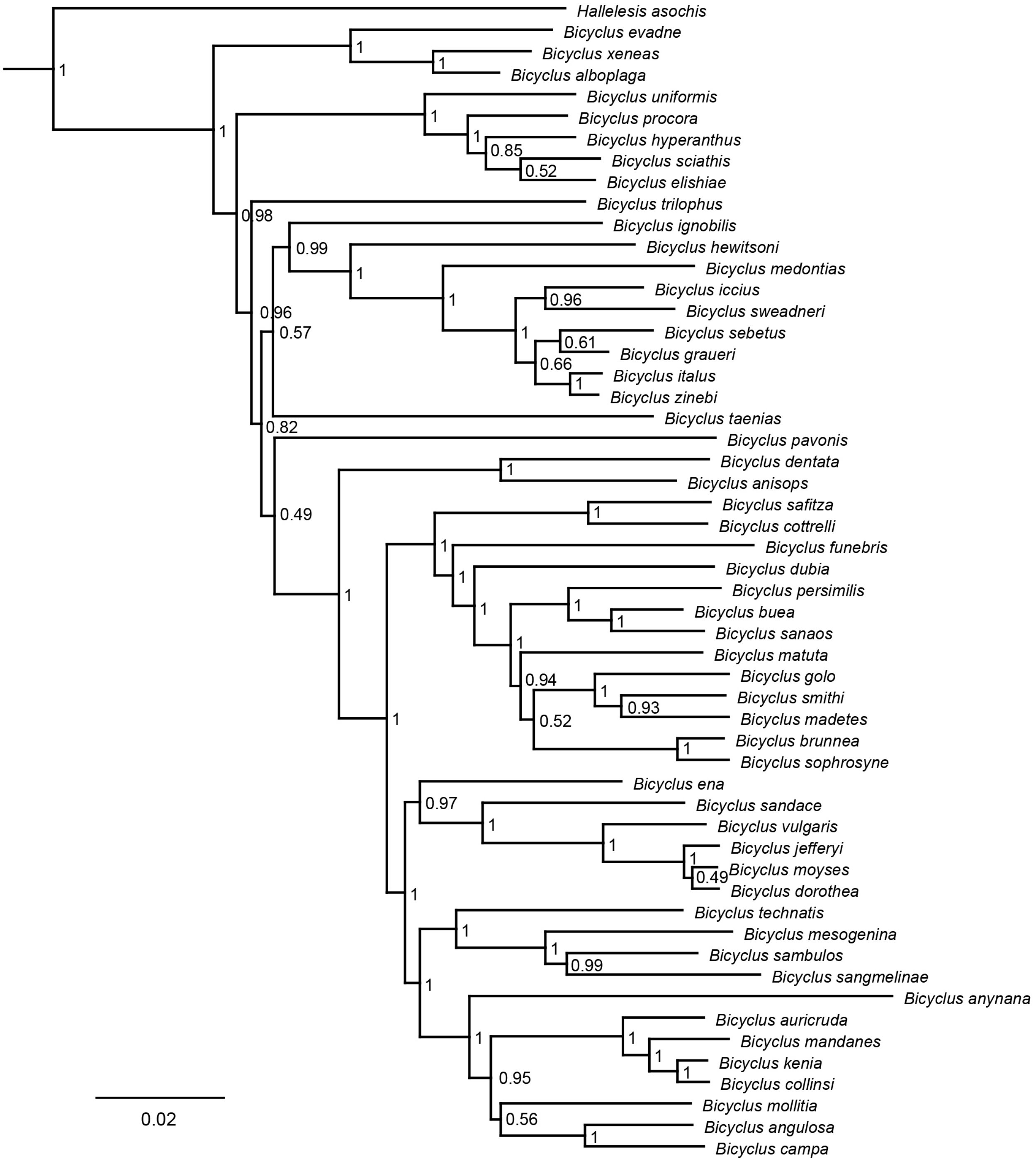

Supplementary Figure 1: Majority-rule consensus bayesian phylogenetic tree of 53 species of the *Bicyclus* genus with *Hallelesis* as the outgroup. The tree was generated in MrBayes v3.2.7a. Numbers at the node indicate posterior probabilities.

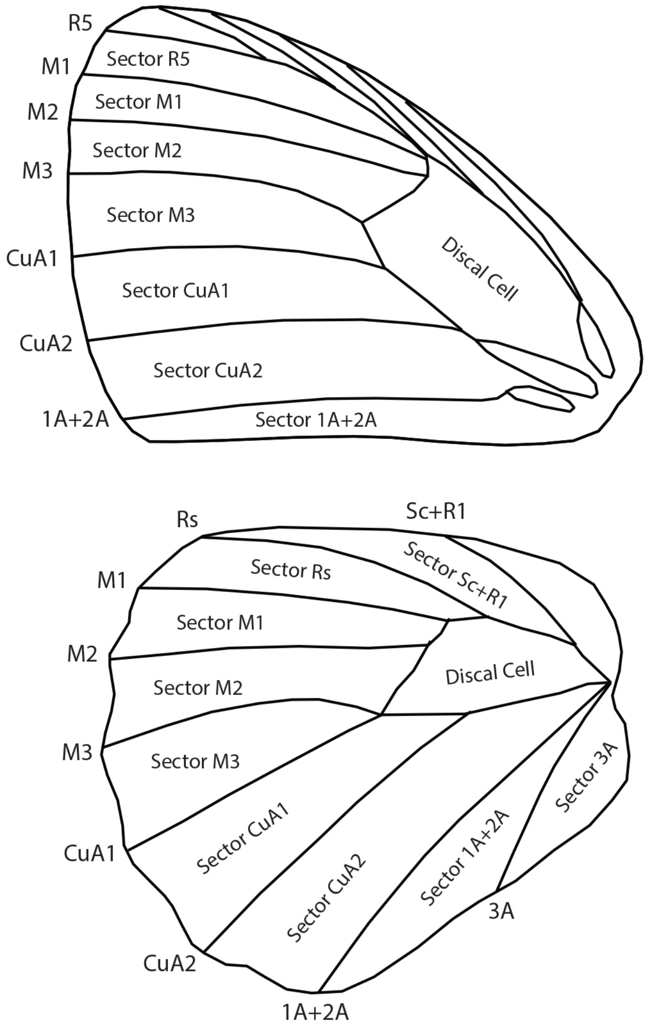

Supplementary Figure 2: Notation of wing sectors and veins on *Bicyclus anynana* wings. The names of the sectors (within the wing) and veins (outside the wing) used to reference the different locations on the wing disc is shown.

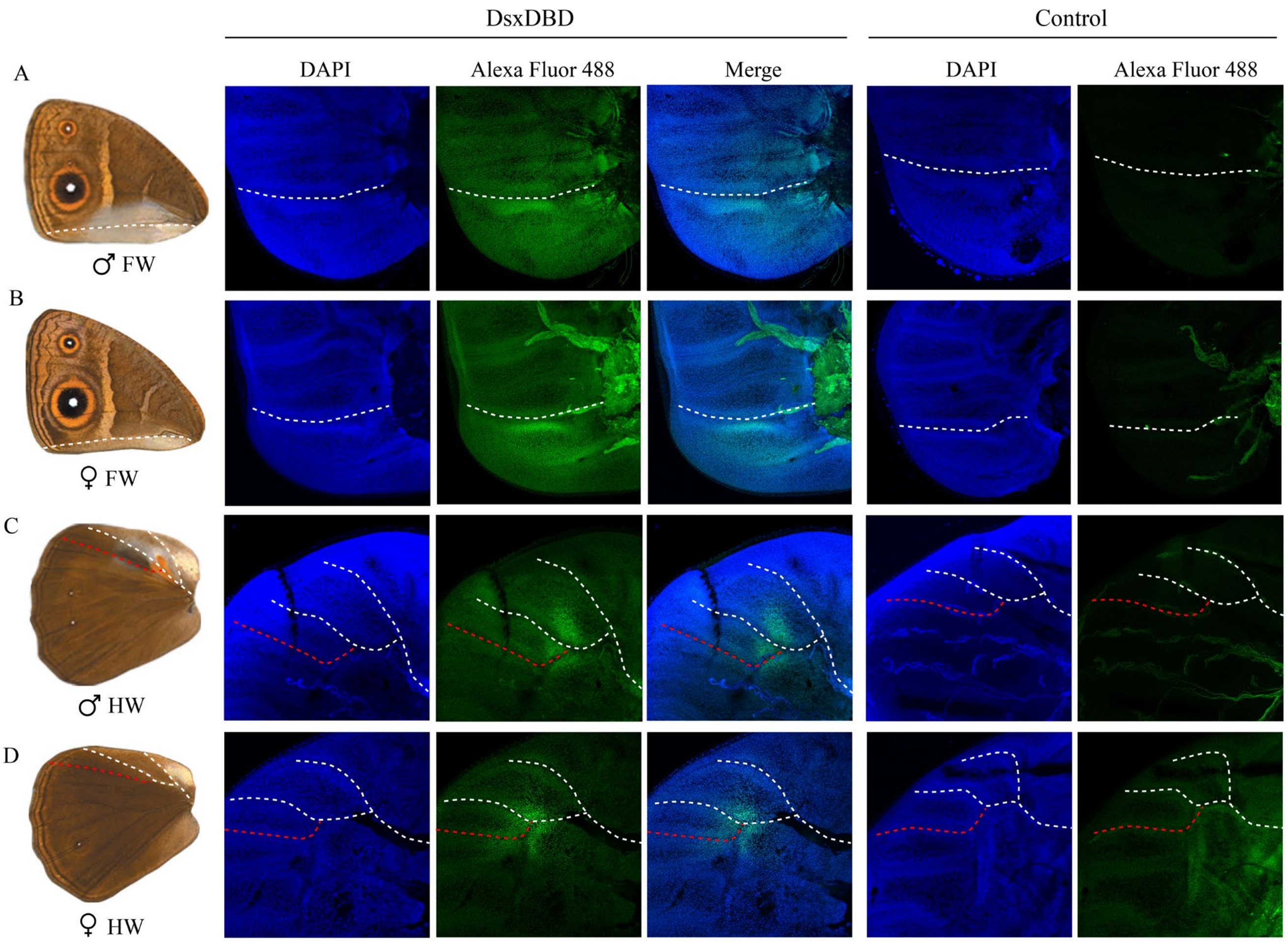

Supplementary Figure 3: Co-immunostainings of *Bicyclus anynana* wing discs with DAPI and Dsx-DBD antibody during the Mid-5th instar larval stage. Expression is shown for (A) male and (B) female forewings and (C) male and (D) female hindwings. Controls stains with DAPI and secondary antibody only are shown in the last two columns. Images are the best, illustrative images. White and red dotted lines denote homologous veins in the respective wings**.**

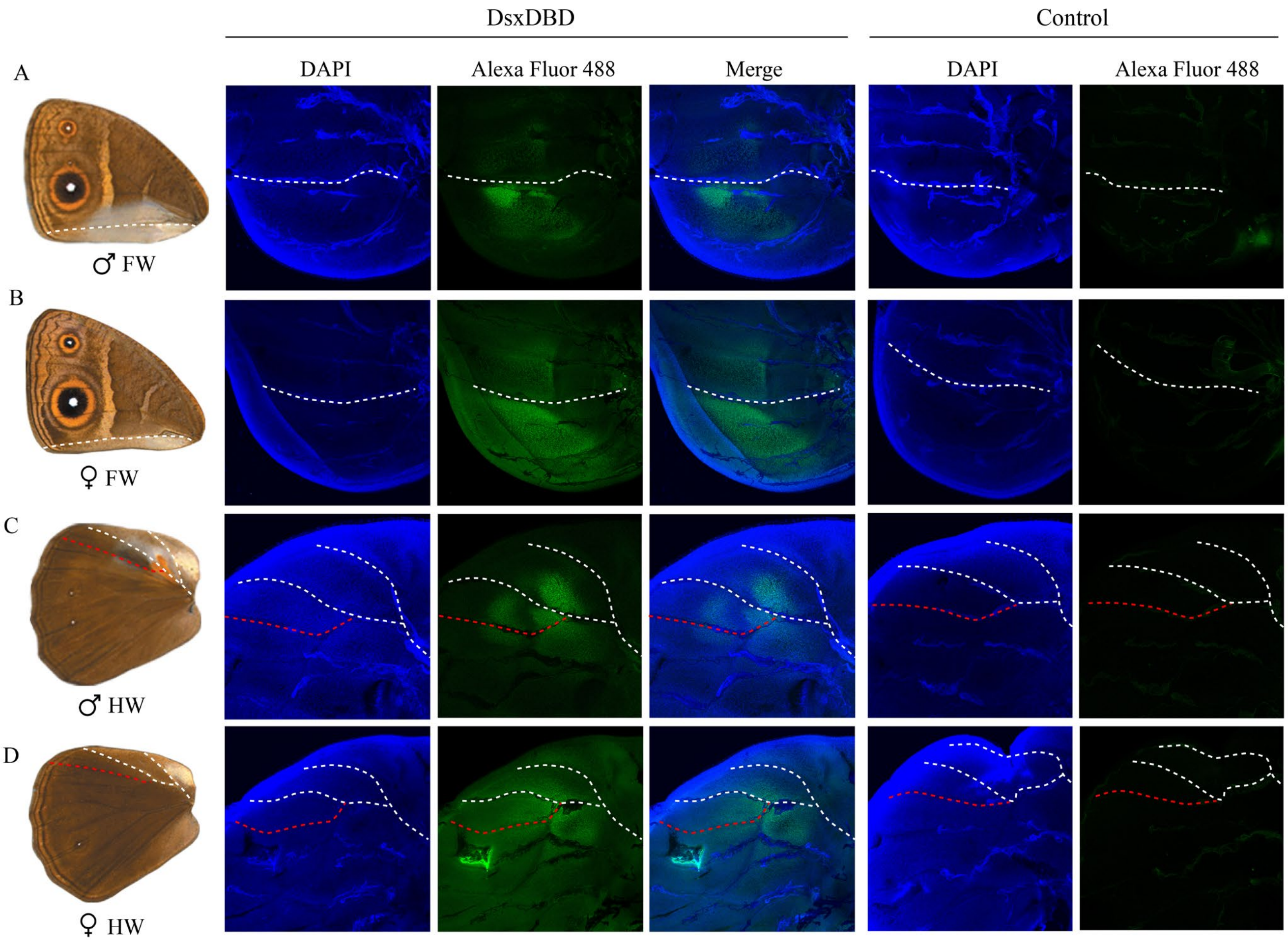

Supplementary Figure 4: Co-immunostainings of *Bicyclus anynana* wing discs with DAPI and Dsx-DBD antibody during the wandering stage. Expression is shown for (A) male and (B) female forewings and (C) male and (D) female hindwings. Controls stains with DAPI and secondary antibody only are shown in the last two columns. Images are the best, illustrative images. White and red dotted lines denote homologous veins in the respective wings**.**

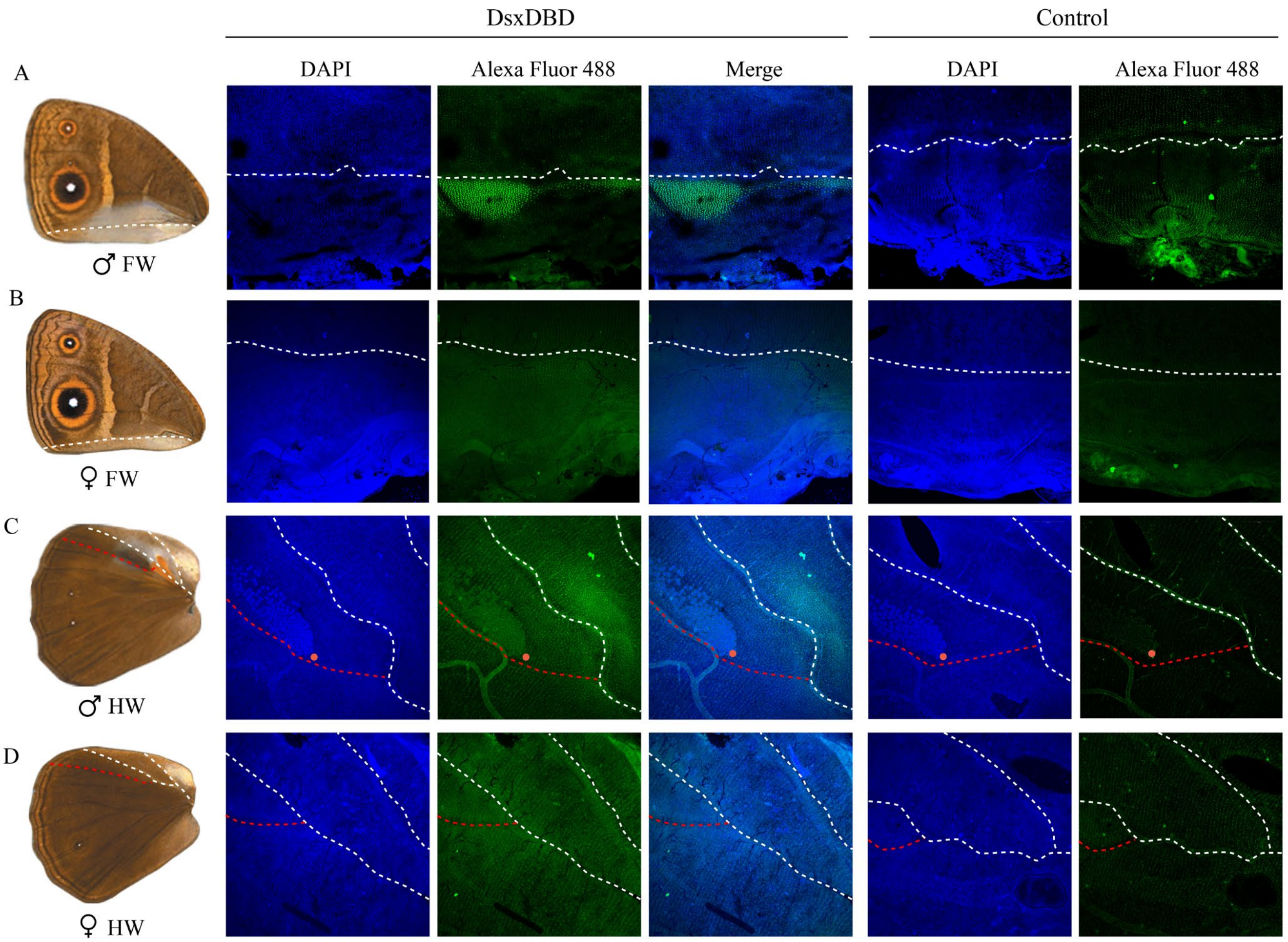

Supplementary Figure 5: Co-immunostainings of *Bicyclus anynana* wing discs with DAPI and Dsx-DBD antibody ~28 hours after pupation. Expression is shown for (A) male and (B) female forewings and (C) male and (D) female hindwings. Controls stains with DAPI and secondary antibody only are shown in the last two columns. Images are the best, illustrative images. White and red dotted lines denote homologous veins in the respective wings and the orange dots in (C) correspond to the base of hair-pencil 2 as in Figure 1.

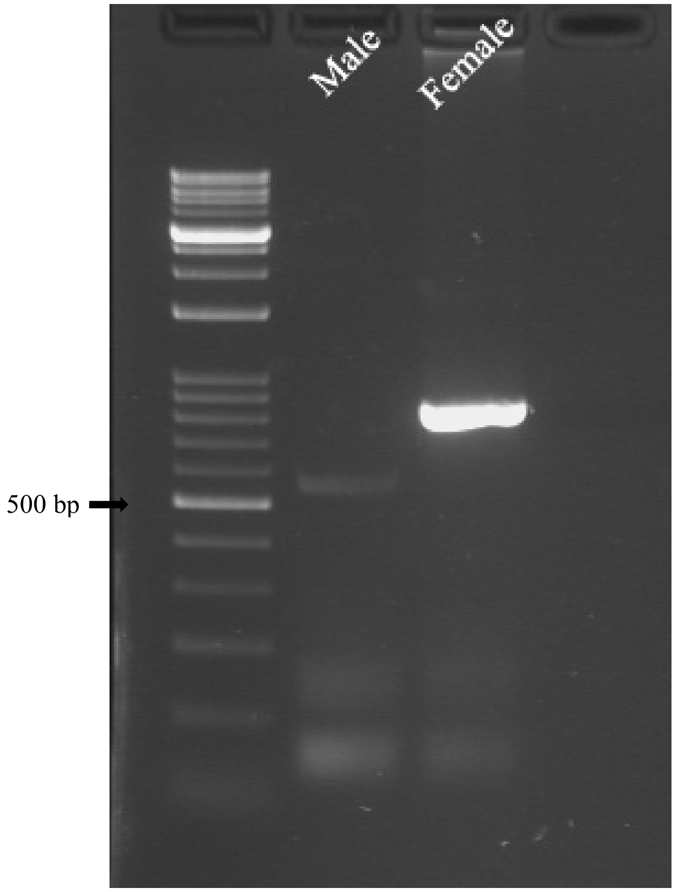

Supplementary Figure 6: PCR amplification of *dsx* from ~18 hr pupal wing tissues of both sexes of *Bicyclus anynana*. The wing tissues of each sex express sex-specific isoforms of *dsx*. The partial sequences generated are available on GenBank (accession nos. MK869725 and MK869726).

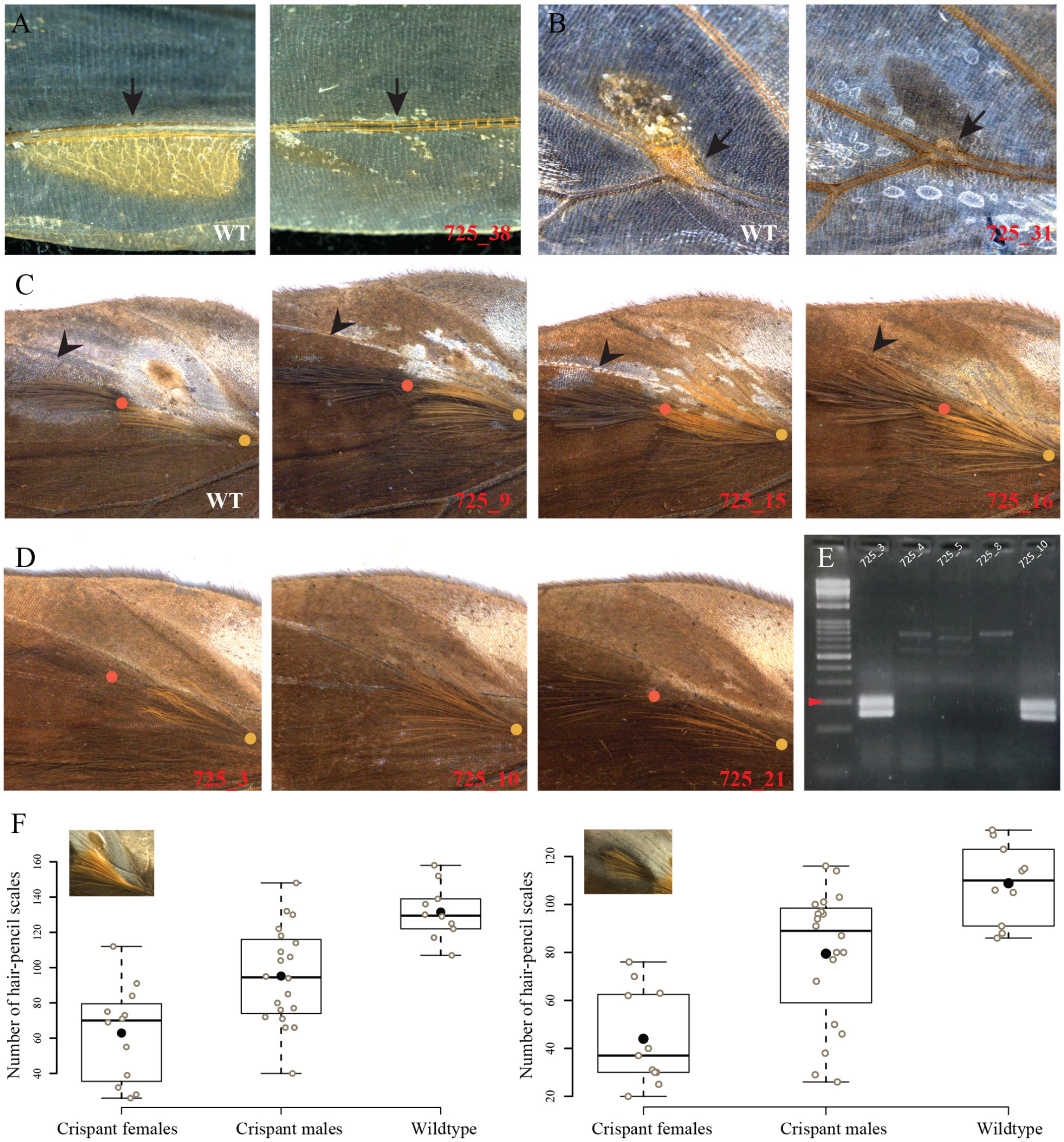

Supplementary Figure 7: CRISPR/Cas9 mediated *dsx* crispant phenotypes and verification of genetic sex. (A,B) Loss of patch-associated glands in crispant males (black arrows) in comparison to wildtype (WT) C) Hindwing scent organs of male crispant individuals (in red) compared to WT. Black arrowheads indicate affected development of the greyish-silver scales of patch 2 that lie beneath the black hair-pencil with respect to WT. D) Ectopic hair-pencil phenotypes on female hindwings. The yellow and orange dots in (C) and (D) mark the base of hair-pencils as per Figure 1. E) Amplification of female-specific W-microsatellite (~200bp, red arrowhead) to verify the sex of the mutants. Lane 1 is the DNA ladder. Samples 725_3 and 725_10 are females, the others are male. (F) Counts of hair-like scales in hair-pencil 1 (left) and hair-pencil 2 (right) in crispant females, crispant males, and WT. The means between the different groups are different according to a one-way ANOVA and all comparisons are significantly different from each other (adj $p<0.05$, Tukey's HSD). Inset shows the hair-pencil considered for each set of graphs.

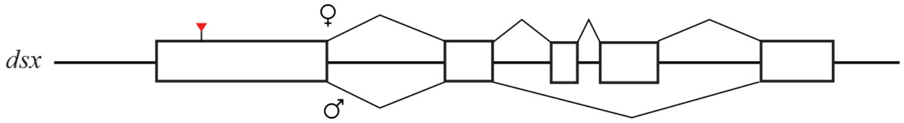


```
WT     GAGCTGAAGGGCCACAAG------------------------------------CGGTACTGCAAGTACCGGTACTGCACGTG
725_3  GAGCTGAAGGGCCACAAG----------------------------------GGCGGTACTGCAAGTACCGGTACTGCACGTG
725_3  GAGCTGAAGGGCCACAAGCTTAGCCGGTACTTGCAGTACCGGTACTTGCAGTACCGGTACTGCAAGTACCGGTACTGCACGTG
725_3  GAGCTGAAGGGCCACAAG---------------------------------------TACTGCAAGTACCGGTACTGCACGTG
725_4  GAGCTGAAGGG----------------------------------------------TACTGCAAGTACCGGTACTGCACGTG
725_4  GAGCTGAAGGGCCACAAG------------------------------------------------TACCGGTACTGCACGTG
725_5  GAGCTGAAGGGCCA--------------------------------------------ACTGCAAGTACCGGTACTGCACGTG
725_5  GAGCTGAAGGGCCACAAG-----------------------------------GCGGTACTGCAAGTACCGGTACTGCACGTG
725_5  GAGCTGAAGGGCCACAAG--------------------------------TACGCGGTACTGCAAGTACCGGTACTGCACGTG
725_5  GAGCTGAAGGGCCACAAG---------------------------------------TACTGCAAGTACCGGTACTGCACGTG
725_8  GAGCTGAAGGGCCACAAG------------------------------------CGGTACTGCTAGTACCGGTACTGCACGTG
725_8  GAGCTGAAGGGCCAC----------------------------------------GGTACTGCAAGTACCGGTACTGCACGTG
725_8  GAGCTGAAGGGCCACAAG------------------------------TCACAGCGGTACTGCAAGTACCGGTACTGCACGTG
```

```
WT     ELKGHK------------RYCKYRYCTCEKCRLTADRQRVMALQT
725_3  ELKGHK--------------GGTASTGTARARSAG*RPTGSGSWR
725_3  ELKGHKLSRYLQYRYLQYRYCKYRYCTCEKCRLTADRQRV-----
725_3  ELKGHK-------------YCKYRYCTCEKCRLTADRQRVMAL*T
725_4  ELKGTA------------------STGTARARSAG*RPTGSGSWR
725_4  ELKGHK----------------YRYCTCEKCRLTADRQRVMALQT
725_5  ELKGQL---------------QVPVLHVREVPADGRPAAGHGAAD
725_5  ELKGHK------------AVLQVPVLHVREVPADGRPAAGHGAAD
725_5  ELKGHK-----------YAVLQVPVLHVREVPADGRPAAGHGAAD
725_5  ELKGHK-------------YCKYRYCTCEKCRLTADRQRVMALLT
725_8  ELKGHK------------RYC*YRYCTCEKCRLTADRQRVMALQT
725_8  ELKGHG----------------TASTGTARARSAG*RPTGSGSWR
725_8  ELKGHK----------SQRYCKYRYCTCEKCRLTADRQRVMALQT
```

Supplementary Figure 8: Sequence genotyping of *dsx* crispants in *Bicyclus anynana*. Top: Schematic of *dsx* male and female isoforms with the region targeted by CRISPR/Cas9 indicated with a red marker. Boxes are the different exons. Bottom: Nucleotide sequences and their translated amino acid sequences from the targeted region of crispant individuals compared with the wildtype sequence in bold. Blue is the target sequence and the PAM sequence is in red. Red stars in the translated protein sequences indicate truncated proteins due to stop codons.

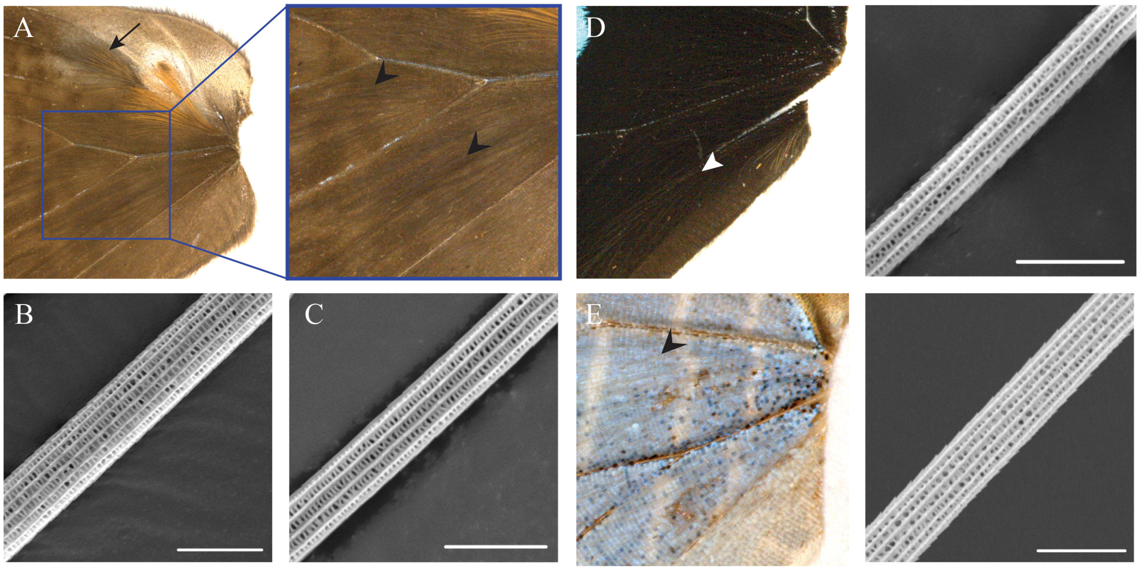

Supplementary Figure 9: Optical and scanning electron micrographs of hair-pencils and long hairs. A) Dorsal hindwing of *B. anynana*. Boxed region is expanded to show the long hair-like scales (black arrowheads) that occur on the wings. Black arrow points to the black hair-pencil. B) Scanning electron micrograph (SEM) of a black hair-pencil of *B. anynana*. C) SEM of a long hair-like scale of *B. anynana*. D) Dorsal hindwing of *Papilio palinurus* (left) and the SEM of a long hair-like scale (right), indicated by the white arrowhead. E) Dorsal hindwing of a lycaenid butterfly (left) and the SEM of a long hair-like scale of this species (right), shown by the black arrowhead. Scale bars: 10µm.

Supplementary Table 2: Comparison of a single-origin vs multiple-origins hypothesis for different forewing and hindwing hair-pencils and patches among *Bicyclus* species. The MRCA (Most Recent Common Ancestor) of all lineages that bear the trait of interest was fossilized to either 1 (trait present, single origin) or 0 (trait absent, multiple-origins). Log marginal likelihoods of the two models were calculated using a ReverseJump MCMC analysis on the 15000 post burn-in trees generated from MrBayes. 2*(Δ log marginal likelihood) is the Log Bayes Factors statistic for model testing and values >2 provide positive support towards the better model, which is highlighted in bold.

**Trait of interest**	**-log marginal likelihood of MCRA = 0**	**-log marginal likelihood of MCRA = 1**	**2*(Δ log marginal likelihood)**
HW Hair-pencil 1	10.7185	**8.66153**	**4.114**
HW Hair-pencil 2	**29.6508**	30.7024	**2.1032**
HW Hair-pencil 3	26.0791	26.6614	1.165
HW Hair-pencil 4	**25.2632**	26.6401	**2.7538**
HW Hair-pencil 6	**11.3398**	13.297	**3.914**
FW Hair-pencil 10	14.4906	**12.2945**	**4.392**
HW Patch 2	30.5968	31.4624	1.7312
HW Patch 3	**24.3597**	27.3627	**6.006**
HW Patch 4	**24.4543**	26.8985	**4.8884**
FW Patch 8	**11.6251**	14.2916	**5.333**
FW Patch 10	26.8476	27.7528	1.8104
FW Patch 11	**12.0818**	13.7735	**3.38**
FW Patch 14	**15.1644**	17.2407	**4.1526**

FW Patch 15	31.3586	30.7791	1.159
FW Patch 16	32.6581	32.9647	0.6132
FW Patch 17	11.8391	**9.5876**	**4.503**

Supplementary Table 3: Comparison of a dependent vs independent model of evolution for different pairs of hair-pencils and patches. Log marginal likelihoods of the two models were calculated using a ReverseJump MCMC analysis on the 15000 post burn-in trees generated from MrBayes. 2*(log marginal likelihood(dependent) – log marginal likelihood (independent)) is the statistic for model testing and values >2 provide support for the dependent model. Pairs of hair-pencils and patches that show correlated evolution are highlighted in bold.

Trait 1	Trait 2	-log marginal likelihood (Dependent)	-log marginal likelihood (Independent)	2*(Δ log marginal likelihood)
**HW Hair-pencil 1**	**HW Patch 1**	11.5414	15.3335	**7.60**
**HW Hair-pencil 2**	**HW Patch 2**	46.5223	58.1527	**23.26**
**HW Hair-pencil 3**	**HW Patch 3**	38.8432	48.8338	**19.98**
**HW Hair-pencil 4**	**HW Patch 4**	37.1683	47.9479	**21.56**
**HW Hair-pencil 1**	**FW Patch 15**	37.9627	39.4948	**3.064**
HW Hair-pencil 1	FW Patch 16	41.7061	41.2099	-0.9924
HW Hair-pencil 2	FW Patch 15	59.9011	58.7345	-2.33
**HW Hair-pencil 2**	**FW Patch 16**	51.5141	60.3556	**17.683**

Supplementary Table 4: Primers and guide RNA sequences used in this study

Gene	Primer Number	Primer Sequence
dsx isoform sequencing	AM 27<br>AM 1118	Forward 5’ ACTGCACGTGCGAGAAGTG 3’<br>Reverse 5’ GAGCAGCACACGCCGTAC 3’
dsx common CRISPR Guide	AM 725	5’GAAATTAATACGACTCACTATAGGTACTTGCAGTACC GCTTGGTTTTAGAGCTAGAAATAGC 3’
dsx common Genotyping	AM 695<br>AM 696	Forward 5’ GCAATCATCAGTCGCATCGTG 3’<br>Reverse 5’ CAAGTGTGGGAACAGCTAAAG 3’
W-microsatellite primers for sexing *Bicyclus anynana*	AM 797<br>AM 798	Forward 5’ GAACCAATGCGACAAATGCGACAT 3’<br>Reverse 5’ TGATCTGTATAATCTAATCATAGTGGGTAT AACTAAAACT 3’

Supplementary Table 5: CRISPR/Cas9 injection concentrations and mutation frequencies

Guide	Guide RNA Conc (ng/µl)	Cas9 mRNA Conc (ng/µl)	Eggs injected	Eggs hatched	Hatch ratio	Total adults	Mutant phenotypes
dsx common	400	900	420	166	39.52 %	83	32 (38.55%)

Supplementary Table 6: Quantification of male and female crispants showing different types of *dsx* crispant phenotypes.

**Type of *dsx* mutant**	**Number of males (n=20)**	**Number of females (n=12)**
**Appearance of hair-pencils**		12
**Reduction in length of white band**		9
**Loss of ventral forewing androconia**	20	
**Loss of dorsal hindwing androconia**	19	
**Visible reduction in hair-pencil number**	5	
**Intersex genitalia phenotype**	10 (12 individuals were examined)	8 (8 individuals were examined)
